# Experimental studies on squeezing interstitial fluid via transfer of ultrasound momentum (SIF-TUM) in *ex vivo* chicken and porcine tissues

**DOI:** 10.1063/5.0235806

**Published:** 2025-04-01

**Authors:** Liqin Ren, Na Thi Vy Nguyen, Tingfeng Yao, Kytai T. Nguyen, Baohong Yuan

**Affiliations:** 1 Ultrasound and Optical Imaging Laboratory, Department of Bioengineering, The University of Texas at Arlington, Arlington, Texas 76019, USA; 2 Joint Biomedical Engineering Program, The University of Texas at Arlington and The University of Texas Southwestern Medical Center at Dallas, Dallas, Texas 75390, USA; 3 Department of Bioengineering, The University of Texas at Arlington, Arlington, Texas 76019, USA

## Abstract

The ultrasound-assisted transport of drugs or fluorophore-loaded nanoagents plays an important role in the desirable drug delivery and imaging contrasts. Unlike conventional ultrasound techniques that rely on thermal or cavitation effects, this study aims to conduct an experimental investigation into the dynamics of interstitial fluid streaming and tissue recovery in *ex vivo* chicken breast and porcine loin muscle tissues during and after ultrasound exposures, which has not been experimentally investigated in the literature. Biological tissues consist of both a fluid and a solid matrix, and an ultrasound beam compresses the tissues within a small focal volume from all directions, which generates macroscopic streaming of interstitial fluid and compression of the tissue's solid matrix. After the ultrasonic exposure, the solid matrix undergoes recovery, leading to a backflow of the fluid matrix. Temperature-insensitive sulforhodamine-101 encapsulated poly(lactic-co-glycolic acid) nanoparticles with an average diameter size of 175 nm were locally injected into *ex vivo* chicken breast and porcine loin muscle tissues to study the ultrasound-induced dynamics in the tissues during and after ultrasound exposure by analyzing the distribution of fluorescence. The changes in fluorescence over time caused by the streaming and backflow of interstitial fluid were studied with two *ex vivo* tissue models, and a faster recovery was observed in porcine tissues compared with chicken tissues. The ultrasound-induced transportability of the nanoagent in porcine muscle tissues was much higher (∼8.75 times) than in chicken breast tissue likely due to structural differences. The study reveals a promising, non-invasive strategy for enhancing drug delivery in dense tissues by leveraging mechanical ultrasound effects, potentially advancing therapeutic and diagnostic applications.

## I. INTRODUCTION

Nanotechnology has been extensively explored for cancer diagnosis and treatments in the past decades.^1^ However, the drug delivery efficiency is still not satisfactory, and only 0.7% of administered nanoparticles entered solid tumors based on the studies published from 2005 to 2015.^2^ The clearance from the bloodstream by the immune, reticuloendothelial, and renal systems, limited diffusion in the dense extracellular matrix, and elevated interstitial pressure gradients pose a significant challenge to effective agent delivery via nanoparticles.^3,4^

Ultrasound-assisted drug delivery has been shown to enhance the mobility of therapeutic or diagnostic agents within diseased tissues and increase permeation across the blood–brain barrier.^5,6^ Several mechanisms of ultrasound-enhanced drug delivery have been proposed, including mechanical effects (such as sonoporation, cavitation, microstreaming, and agent-enhanced effects via microbubbles or pressure-sensitive proteins) and thermal effects (hyperthermia, diffusion, and thermophoresis).^7–11^ For example, non-thermal effects presenting in low-intensity pulsed ultrasound exposure can contribute to the release of anticancer drugs from loaded block co-polymer nanoparticles.^12^ This has been achieved through shear stress and shock waves from the bubble collapse during cavitation. On the other hand, the thermal effects are due to the absorption of acoustic energy in the tissues using high-power continuous or pulsed ultrasound.^13^

However, it is still challenging to fully understand the interactions between nanoparticles and mechanical forces generated by non-thermal low-intensity pulsed ultrasound waves, especially in deep tissues. Recently, a new macroscopic streaming mechanism, named squeezing interstitial fluid via transfer of ultrasound momentum (SIF-TUM), has been proposed and modeled by our lab to explain possible ultrasound-driven nanoparticle motion enhancement in deep tissues.^14^ Briefly, a tissue is considered a two-phase material, including both interstitial fluid (movable) and solid matrix (deformable). When an ultrasound beam is tightly focused on the tissues, it can pass its momentum to tissue interstitial fluid and push the fluid flowing out of the ultrasound focal volume from all directions. This generates a macroscopic streaming of the interstitial fluid (away from the focal volume). At the same time, this results in the inward compression of the solid tissue matrix around the focal volume if no void space can exist in the tissue. After the ultrasound exposure, the solid matrix undergoes recovery (i.e., expanding back to the original position), and, therefore, the interstitial fluid must flow back into the ultrasound focal volume, which can bring the nanoparticles back too. In addition, the thermal effects, such as thermophoresis via temperature gradient and diffusion via concentration gradient, on the particle motion were found much weaker than the streaming caused by SIF-TUM.^14^

We hypothesize that the transfer of ultrasound momentum (SIF-TUM) can induce macroscopic streaming effects in *ex vivo* tissues, driving the motion of nanoparticles through interstitial spaces and enabling their recovery after ultrasound exposure. As claimed in the previous study,^14^ streaming occurring at the tissue-and-water-bath boundary (used in the literature) may not fully reflect the dynamics of SIF-TUM and may not simulate real applications in deep tissues. In this study, we investigated streaming inside tissues by focusing an ultrasound beam 2–3 mm away from the top boundary between the tissue and air interface. In addition, to avoid the confounding between mechanical and thermal effects, we selected a temperature-insensitive fluorophore, sulforhodamine 101 (SR101), and encapsulated it into a temperature-insensitive nanoparticle, made of poly(lactic-co-glycolic acid) (PLGA). We denoted this nanoparticle as SR101-PLGA and injected them locally in *ex vivo* animal tissues. Using the same system as our ultrasound-switchable fluorescence (USF) imaging,^15–17^ we studied the dynamics of the ultrasound-induced motions of the nanoparticle (SR101-PLGA) in both chicken breast and porcine loin muscle tissues. We found that ultrasound-caused fluorescence reductions and recoveries were clearly noticed within the ultrasonic focal area in two types of tissues and in agreement with our theoretical studies.^14^ The dynamic fluorescence changes are tissue-dependent, with a faster recovery in porcine than in chicken tissues. These results not only experimentally demonstrate the SIF-TUM mechanisms but also may benefit the researchers who are looking for an accelerated and controlled distribution of nanoagents with the aid of focused ultrasound (FU).

## II. METHODS

### A. Material and synthesis of nanoparticles

To select a temperature-insensitive fluorophore, the following four dyes were tested. Fluorescein 548 (FL548; peak excitation: 498 nm; peak emission: 517 nm) was purchased from Exciton, Inc. (Lockbourne, OH). SR101 sodium salt (peak excitation: 586 nm; peak emission: 606 nm) was purchased from Assay Biotech (Fremont, CA). Cyanine 5 (CY5 NHS ester; peak excitation: 646 nm; peak emission: 662 nm) was from BroadPharm Inc. (San Diego, CA). Indocyanine green (ICG, peak excitation: 789 nm; peak emission: 814 nm) was purchased from Chem-Impex Int'L Inc., USA. Temperature-insensitive polymer, PLGA, lactic/glycolic (LG = 50:50, Mn 15 000–25 000 Da), was purchased from AKina Inc. (West Lafayette, IN). Polyvinyl alcohol (PVA, 15 000–25 000) was purchased from Sigma-Aldrich (St. Louis, MS).

Nanoparticles were synthesized using the standard emulsion method as previously described.^18–20^ Briefly, SR101 was dissolved in 100 *μ*l methanol and was added dropwise to 3 ml of organic solution of chloroform containing 100 mg PLGA. The solution was later added dropwise to 20 ml of the aqueous phase of 5% w/v PVA. The emulsion was probe-sonicated at 25 W for 1 min on ice. The nanoparticle suspension was stirred at 700 rpm and evaporated overnight at room temperature to remove organic solvent. The nanoparticles were collected by ultracentrifugation at 17 000 rpm for 20 min at 4 °C. The nanoparticles were then lyophilized and stored at −20°C after being washed. The freeze-dried sample was resuspended in de-ionized water followed by shaking and vortexing until there was no solid component in the solution for experimental use.

### B. Characterization of fluorophores

The fluorescence vs temperature profiles of various dyes and SR101-PLGA were obtained using a temperature-controlled spectrometer-based system.^16^ The fluorescence level at each temperature was represented by the sum of fluorescence counts in their corresponding emission spectra.

The size of SR101-PLGA was measured by a dynamic light scattering instrument (NanoBrook 90PlusPALS, Brookhaven Instruments, USA) at room temperature. The average diameter size of SR101-PLGA is 174.88 ± 1.83 nm with a polydispersity index of 0.118. The zeta potential of PLGA nanoparticles was estimated to −20.2 ± 1.16 mV according to the previous published work where a similar synthesis method of PLGA nanoparticles was adopted.^18^

### C. H&E staining and image acquisition

Porcine and chicken tissues were embedded in optimal cutting temperature (OCT) compound and flash frozen using liquid nitrogen. Frozen tissues were cross-sectioned at 7 *μ*m thickness using a cryostat (Leica CM1860 UV, Leica Biosystems, Nussloch, Germany) at −20 °C. The sections were hematoxylin and eosin (H&E)-stained, mounted using Permount (SP15-500, Fisher, Fair Lawn, NJ), and then imaged under a bright field microscope (RV2-K, Echo, San Diego, CA).

### D. USF imaging system: *Ex vivo* tissues model

**FIG. 1.**
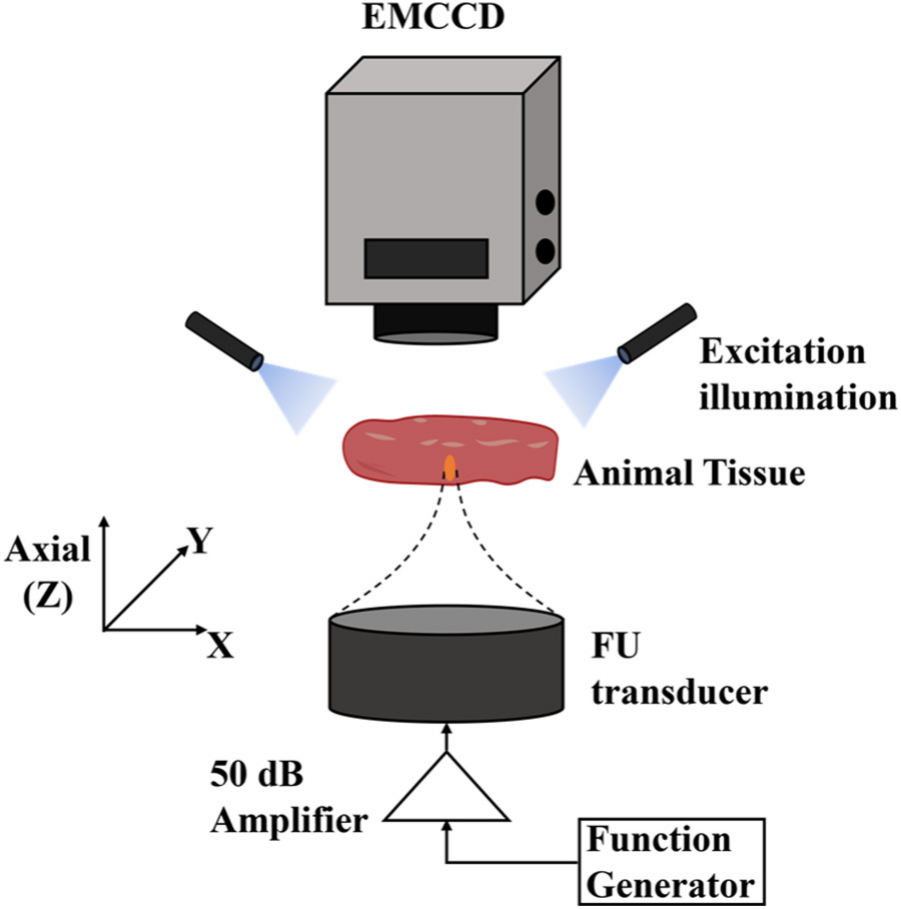
The diagram of the experimental setup. The sample was exposed to a 2.5 MHz FU transducer, and the fluorescence was acquired by an EMCCD camera.

The setup of the USF imaging system has been reported previously, and the schematic diagram is shown in Fig. 1.^15,17^ A 2.5 MHz focused ultrasound (FU) transducer (focal length: 50.8 mm; H-108, Sonic Concepts Inc, USA) was used to emit focused ultrasound waves. The sample was placed in a small tank half-submerged in a big water tank. The fluorescence from the sample passed through three emission filters (BLP01-830R50/25, Semrock Inc., USA) and was captured with an electron multiplying charge-coupled device (EMCCD, ProEM^®^-HS: 1024BX3, Princeton Instruments, USA) equipped with a lens (AF NIKKOR 50 mm f/1.8D Lens, Nikon, Japan). A 473 nm laser (MBL-III-473–20 mW, Dragon Lasers, China) with an intensity of 700 *μ*W cm^−2^ was used as an excitation source.

The frame rate of the EMCCD was 25 Hz, and the EM gain was set to 3. Frames were captured continuously to acquire the temporal profile of fluorescence distribution in the focal area. The first five frames obtained from the camera before the FU exposure were averaged and used as the background fluorescence image. Each 2D frame obtained after the initiation of FU exposure was processed by subtracting the image from the corresponding pixel values in the background fluorescence image. The fluorescence level of each frame was then represented by the average of pixel values in the target area (∼0.7 × 1.0 mm^2^), which is slightly larger than the focal area of FU because the displacements of agents are considered to be accumulative and becoming larger with time. The experiment was conducted with different ultrasound parameters, and each condition was repeated three times, with the curve plotted as the average at each time point.

### E. Samples

150 *μ*L PLGA-SR101 suspension (35 mg/ml) was injected into the tissue samples (chicken breast and porcine loin tissues purchased from local grocery stores) with a thickness of 13 mm via a 31-gauge needle. To avoid possible leakage from the hole created by the needle, instead of from the top surface where the camera field view is located, the nanoparticle solution was injected slowly from one side of the sample (i.e., horizontally injecting the particle solution). The needle tip is ∼2–3 mm away from the top surface. On the other hand, to minimize the boundary effect, the ultrasound beam was focused inside the sample 2–3 mm away from the tissue-and-air boundary.

### F. Data fitting models

The results of the fluorescence reductions during ultrasonic exposures were fitted with a linear fitting model. The recovery segments were fitted using three different models including single-phase exponential regression, two-phase exponential regression, and a customized model. The single-phase and two-phase exponential regression models were selected for their simplicity and effectiveness in capturing the dynamics of fluorescence recovery. The two-phase model, in particular, aligns well with the experimental observations, potentially representing the sequential processes of interstitial fluid streaming and tissue matrix recovery. Curve fitting was conducted in GraphPad Prism (GraphPad Software Inc., San Diego, CA, USA) or using the curve-fitting toolbox in MATLAB (MathWorks, Natick, Massachusetts, USA). Data were smoothed with a gaussian-weighted moving average filter (sliding window = 280 ms) before fitting with the custom *M* model.

In the single-phase exponential model, the fluorescence dynamics $\left(S_{t}\right)$ can be expressed by the following equation:

$$S_{t}=\left(S_{0}-\mathrm{Plateau}\right)e^{-\frac{t}{\tau_{r}}}+\mathrm{Plateau}.$$

The double exponential model can be presented by

$$S_{t}=\left(S_{0}-\mathrm{Plateau}\right)\left(\mathrm{Fast}\text{\%}\right)e^{-\frac{t}{\tau_{\mathrm{fast}}}}+\left(S_{0}-\mathrm{Plateau}\right)\left(1-\left(\mathrm{Fast}\text{\%}\right)\right)e^{-\frac{t}{\tau_{\mathrm{slow}}}}+\mathrm{Plateau},$$

where $S_{0}$ is the initial fluorescence level when time is zero. Plateau is the fluorescence level at infinite times. Fast% is the fraction of the faster exponential component in the equation. $\tau_{r}$, $\tau_{\mathrm{fast}}$, and $\tau_{\mathrm{slow}}$ are the exponential time constants.

The SIF-TUM models provided in the previous publication^14^ usually need to be numerically solved by assuming several parameters. To avoid the complexity and the assumption of unknown parameters, in this study, we significantly simplified the models by boldly only considering the central location of the ultrasound focal volume. Thus, a similar analytical solution provided in the literature (usually for calculating ultrasound-induced temperature in tissue) can be adopted to derive an approximate solution for describing the fluorescence reduction.^21,22^ Briefly, the normalized USF signal at the central location of the ultrasound focal volume (i.e., r=0) is approximately expressed as the following analytical solution in the custom *M* model:

$$\frac{{S_{\mathrm{USF}}|}_{r=0,\;t>t_{0}}}{\left|{S_{\mathrm{USF}}|}_{r=0,\;t=t_{0}}\right|}\propto \frac{-\beta_{z}^{1/2}\beta_{r}}{\left[\left(4M\left(t-t_{0}\right)+\beta_{r}\right)*{\left(4M\left(t-t_{0}\right)+\beta_{z}\right)}^{\frac{1}{2}}\right]},$$

which can be used as the fitting model to extract *M*. Here, $t_{0}$ is the time when ultrasound exposure ends and *M* is a “diffusion-like” parameter that has a unit of meter^2^/second and represents the transportability of nanoagent in tissues at a macroscopic level;^14^ *t* is time; and $\beta_{r}=2\sigma_{L}^{2}$ and $\beta_{z}=2\sigma_{A}^{2}$ are related to the square of the lateral and axial focal size of the ultrasound beam, respectively. $\sigma_{L}$ can be calculated by dividing the lateral FWHM of the focal zone by a constant of 2.35 and, similarly, $\sigma_{A}$ can be calculated by dividing the axial FWHM of the focal zone by the same constant (note that the lateral and axial FWHMs of 0.55 and 3.5 mm were used in the fitting). It is important to know that this solution is an approximated analytical solution. The disadvantage is that this model is not an accurate solution to represent the fluorescence recovery dynamics. The advantage is that it is very simple, and the data can be quickly fitted to extract the value of *M*. More importantly, *M* is the only unknown parameter. In addition, *M* has a clear physical meaning, representing the nanoparticle transportability.^14^ Note that *M* can be accurately defined and derived from the SIF-TUM models by inserting the Eqs. (1) and (2) into (4) in our previous paper^14^ and can be expressed as $M=R_{f}\left(k/\mu \right)H$, in which (a) tissue permeability *k* (related to tissue porosity), (b) tissue fluid viscosity μ, (c) the retardation factor $R_{f}$ between interstitial fluid and the agent (related to the size difference between tissue pores and nanoagent, and also the surface charge effect between the agent and the adopted tissue), and (d) tissue apparent modulus *H* (related to tissue elasticity). It is impossible to estimate *M* based on these unknown and unmeasurable individual factors. However, *M* can be experimentally measured via the experimental fitting methods.

## III. RESULTS AND DISCUSSION

### A. Comparison of fluorophores

**FIG. 2.**
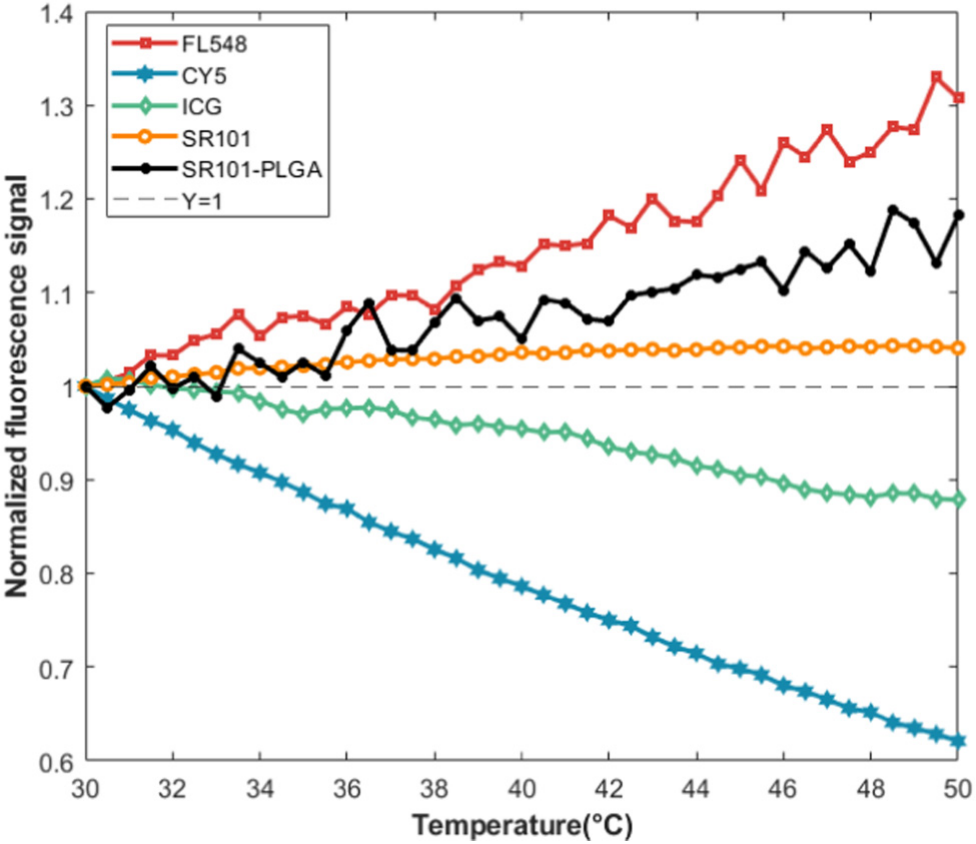
Temperature dependence of the fluorescence emission of FL548 (>561 nm), CY5 NHS ester (>715 nm), ICG (>830 nm), SR101 (>561 nm), and SR101-PLGA (>561 nm) when excited at 473, 640, 808, 473, and 473 nm, respectively.

Figure 2 shows that the fluorescence of various dyes and SR101-PLGA changes with respect to temperature. Fluorescein 548 (FL548) exhibits increased fluorescence over a range of 20 °C with a rate of 1.5% per °C. The fluorescence of cyanine 5 (CY5 NHS ester) and ICG decrease significantly with a rate of 0.6% per °C when the temperature increases from 30 to 50 °C. SR101 grows 0.35% per °C, exhibiting good temperature stability over the temperature range from 30 to 50 °C when dissolved in de-ionized water. SR101-PLGA remains nearly temperature-insensitive and increases 0.91% per °C. Note that the motion of nanoparticles induced by SIF-TUM, causing them to move out of the ultrasound focal volume, results in a reduction of fluorescence within the ultrasound-illuminated region. At the same time, the temperature in the focal volume may slightly rise. The temperature increase caused by the adopted ultrasound has been well discussed in our other studies on USF.^15,16,23,24^ To avoid the interference from the thermal effect, it is better to select a dye that has a positive relationship between fluorescence strength and temperature (such as SR101 or FL548). Thus, the reduction in fluorescence will not be caused by thermal effect, which may not be certain for the dyes that have a negative correlation (such as CY5 or ICG). In this study, we selected SR101, which not only shows a positive relationship but also is relatively less sensitive to temperature than FL548.

### B. Porcine vs chicken comparison

**FIG. 3.**
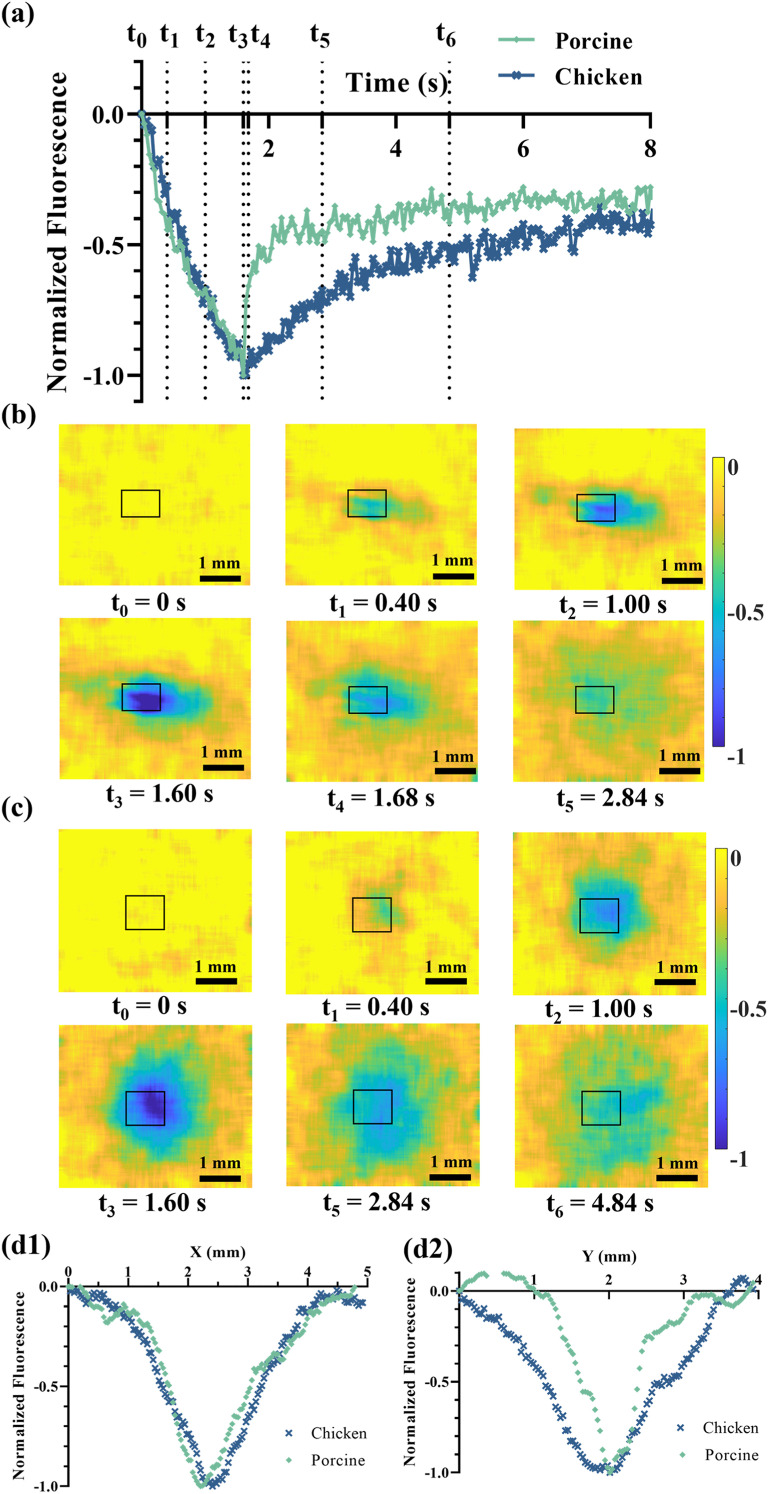
(a) The fluorescence variations with time within the focal area in the animal tissues. The subtracted fluorescence images (5 × 4 mm^2^ view) of (b) porcine tissues and (c) chicken breast tissues were obtained during and after ultrasound exposures. Images were normalized with a 15 × 15 matrix averaging spatial filter. The black box indicates the ROI for calculating the mean fluorescence. Color bars: A value of −1 corresponds to the minimum fluorescence signal observed in the continuously acquired images, while a value of 0 represents the baseline fluorescence signal measured immediately before ultrasound exposure. (d1–2) The spatial profiles (X: horizontal; Y: vertical) of fluorescence image at t_3_.

Figure 3(a) illustrates the temporal profile of the fluorescence in the ROI as indicated by the black box in Figs. 3(b) and 3(c). The fluorescence goes down, showing a similar decay trend in both porcine and chicken tissues when FU is on from 0 to 1.6 s (i.e., from t_0_–t_3_), while fluorescence within the focal volume recovers due to the backflow of agents upon the deactivation of ultrasound. The backflow rate primarily relies on tissue properties (i.e., the parameter *M* discussed above, such as tissue permeability *k*, tissue fluid viscosity μ, the retardation factor $R_{f}$, and tissue apparent modulus H).^14^ *M* represents the capability of agents to flowback into the ultrasound focal volume after ultrasound squeezing via a “diffusion-like” behavior, rather than a concentration gradient. In addition to the parameter *M*, the recovery speed of the fluorescence signal can also be quantified by recovery time constants by fitting the experimental data to an exponential decay function (either a single or multi-exponential decay model), which will be discussed below.

Based on the data in Fig. 3, the recovery of fluorescence in porcine tissues is much faster than that in chicken breast tissues, and a higher initial backflow speed is observed. This may indicate a higher transportability (M), which may be due to higher tissue apparent modulus, a larger pore size in the porcine tissues, and/or lower viscosity of interstitial fluid.^14,25^

Animal tissue is considered to be a biphasic mixture of the solid matrix and liquid matrix, and the nanoparticles are believed to most likely exist in the interstitial fluid due to the administration via the local injection. The backflow rate primarily relies on tissue properties and becomes independent of the ultrasound parameters according to SIF-TUM. Remarkably, the initial backflow rate even surpasses the decay flow rate induced by the ultrasound, suggesting that the fluid containing the fluorescent nanoparticles comes back to the ROI rapidly once the ultrasound illumination stops. However, it quickly diminishes to a small value due to the limited flow velocity of the backflow, which is typically observed in biphasic materials.

A ring-shaped pattern in Figs. 3(b) and 3(c) indicates the outward movements and accumulation of agents in the surrounding region after ultrasound termination. The agents are continuously displaced from the focal center during the ultrasound activation period and back flow immediately after the termination of ultrasound, leading to a recovery of fluorescence level. As nanoparticles are moving toward the surrounding areas of the ultrasound focus, the surrounding fluorescence is expected to increase. However, this may not be visibly obvious in Figs. 3(b) and 3(c) due to the volume effects of surrounding tissues. Figures 3(d1) and 3(d2) present the one-dimensional spatial fluorescence profiles along the horizontal and vertical axes at the moment when FU stopped (i.e., t = t_3_). The vertical spread of the fluorescence patterns in chicken tissues exceeds that observed in pork, while their horizontal dimensions are similar. This discrepancy can likely be attributed to the distinct muscle arrangements in chicken and pork tissues, which, in turn, influence the pathway of ultrasound penetration.

The ultrasound-caused fluorescence decay and the subsequent fluorescence recovery due to the backflow processes were also investigated with different ultrasound strengths (controlled by the applied voltage) and exposure time in both pork and chicken breast tissues. Three trials were repeated in each experiment condition, and a curve was plotted as the average of three sets of results.

**FIG. 4.**
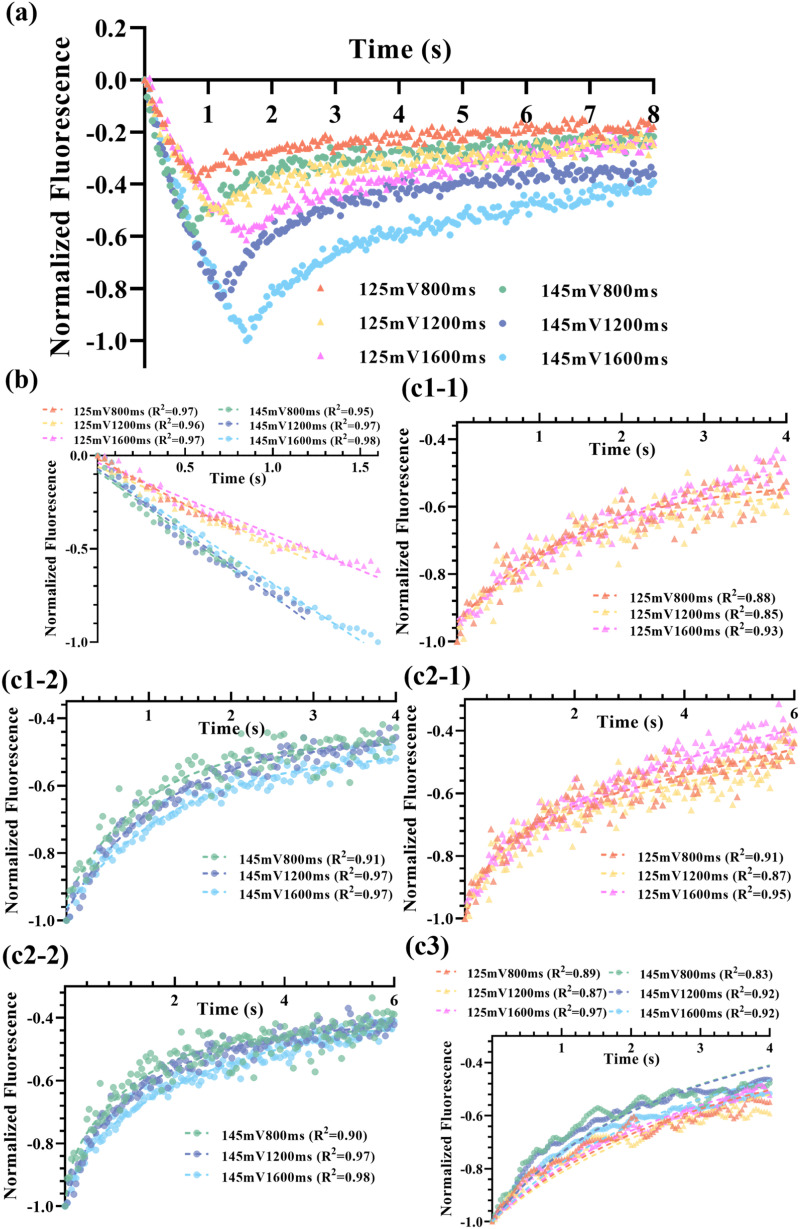
The temporal fluorescence profiles in chicken breast tissues. (a) The fluorescence decreases during ultrasound exposure, and (b) the results were fitted with a linear regression model. (c) The recovery of fluorescence and the results were processed with (c1–1) and (c1–2) single-phase exponential; (c2–1) and (c2–2) double exponential; and (c3) custom *M* equations.

**FIG. 5.**
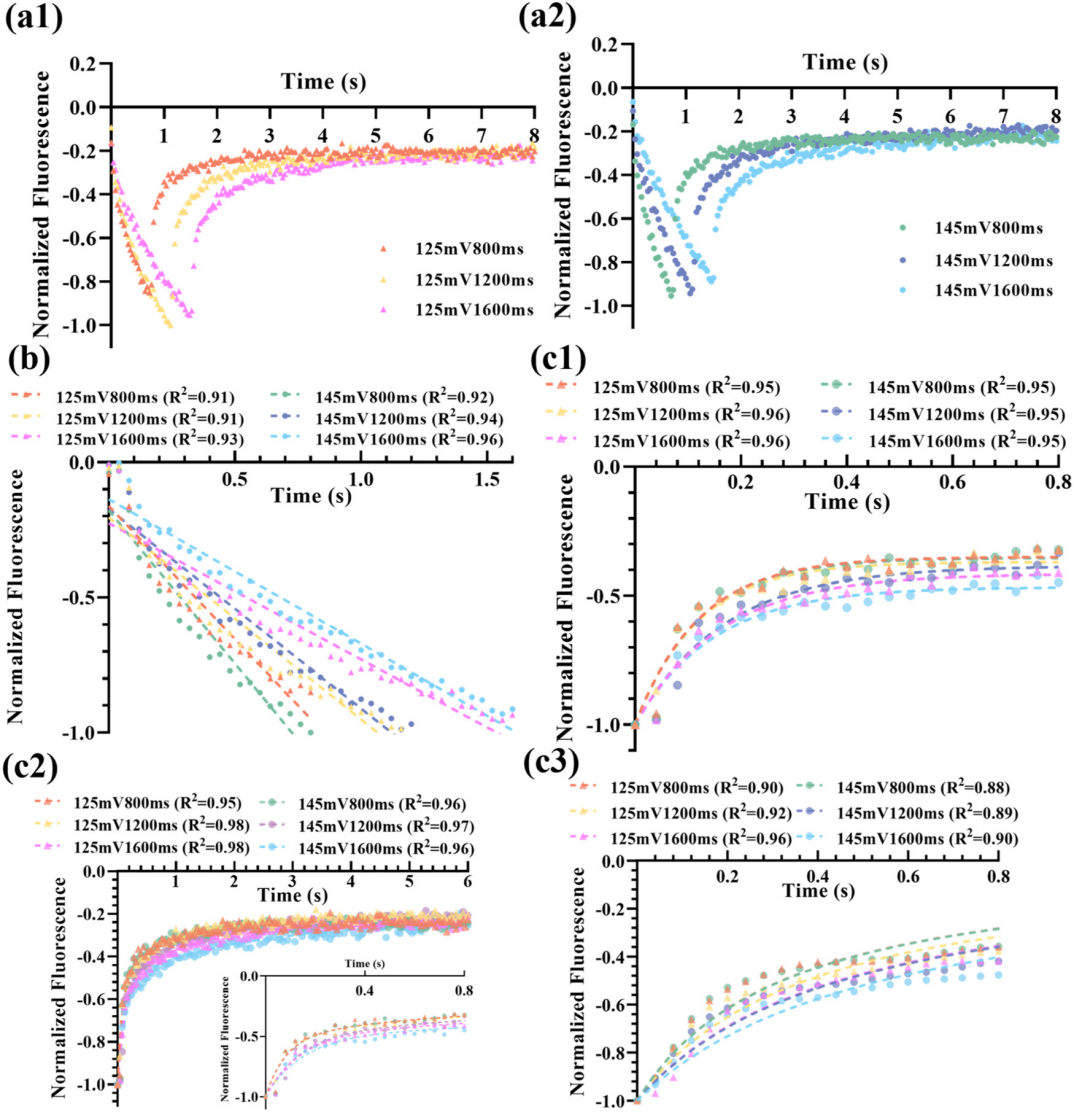
The dynamic fluorescence profiles in porcine tissues for the same voltage at (a1) 125 and (a2) 145 mV. (b) The recovery of fluorescence after FU exposure, and the dashed curves represent the fitted linear results. (c) The recovery of fluorescence and their fitting results with (c1) single exponential and (c2) two-phase exponential; inset: a zoom-in plot within a period from 0 to 0.8 s. (c3) Custom *M* models (dashed curves).

**FIG. 6.**
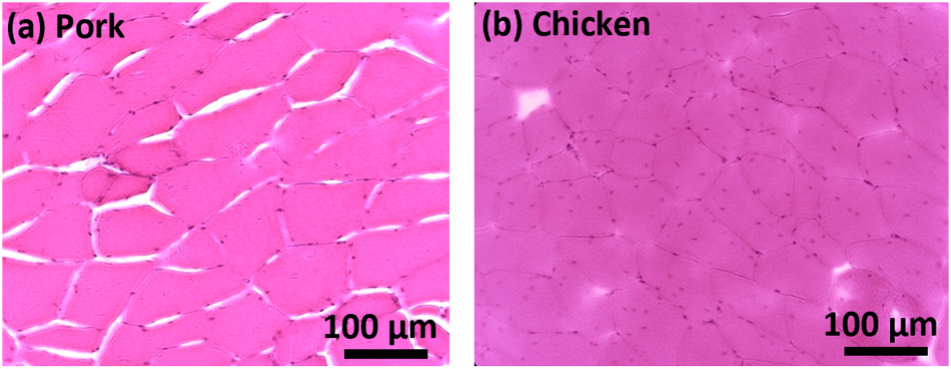
Image of H&E stained microstructure of tissues. (a) Porcine loin muscle; (b) chicken breast.

Figure 4(a) shows the dynamics of the fluorescence reduction and recovery in the chicken breast tissues, respectively. Two voltages (125 and 145 mV) and three exposure times (0.8, 1.2, and 1.6 s) from the function generator were tested. One can find that the fluorescence reduction time is increased with the exposure time, which is understandable because the SIF-TUM effect is directly related to the exposure time. In addition, the extent of the fluorescence reduction is related to both the exposure strength and time. The longer the exposure time and the higher exposure strength can induce higher fluorescence reduction. Specifically, the fluorescence reductions of six groups were normalized in relation to the maximum reduction observed in the 145 mV, 1600 ms group (designated as 100%). The corresponding reductions for the other groups are as follows: 83.6% for the 145 mV, 1200 ms; 61.3% for the 125 mV, 1600 ms; 58.4% for the 145 mV, 800 ms; 51.2% for the 125 mV, 1200 ms; and 38.3% for the 125 mV, 800 ms. This result indicates that higher voltage (i.e., higher ultrasound strength) induces higher streaming velocity of the intestinal fluid to be “squeezed” out of the focal volume [i.e., more negative slopes in Fig. 4(a) for higher voltage]. The results in Fig. 4(a) are consistent with the prediction from the SIF-TUM model.^14^ Figures 5(a1) and (a2) show the results in porcine tissues. Similarly, increasing the exposure time results in a longer fluorescence reduction period, which agrees with the results in Fig. 4(a). However, it does not increase the extent of fluorescence reduction. In addition, increasing the voltage (i.e., increasing the ultrasound strength) does not consistently lead to an increased fluorescence reduction. This phenomenon can be elucidated by considering the possibility of increased tissue fluid loss due to the porosity of the porcine muscle fiber structure, as visually depicted in Fig. 6, especially when multiple experiments are conducted consecutively at the same location. The microstructure of pork exhibits greater porosity with larger intercellular gaps, whereas Fig. 6(b) reveals denser and tighter interconnections among chicken muscle cells.

Figure 4(b) shows the linear fitting of the fluorescence reduction in chicken breast tissues with an R^2^ ≥ 0.95. However, Fig. 5(b) presents that the performance of the linear model is slightly worse in porcine muscle tissues with the lowest R^2^ at 0.91 for two groups (125 mV, 800 ms and 125 mV, 1200 ms). This decrease in R^2^ can be attributed to the faster initial fluorescence reduction during the early stages of ultrasound stimulation. This phenomenon is likely due to the less resistance to the movement of nanoparticles in the more porous tissue structure of porcine samples, resulting in misalignment between the early data points and the linear fitting curves. The fitted time constants for the recovery periods are denoted $\tau_{r}$ and listed in Table I. The single-phase exponential model in porcine muscle tissues as illustrated in Fig. 5(c1) exhibits an excellent fitting performance with the coefficient of determination of R^2^ ≥ 0.95. However, R² is slightly less than 0.9 in the chicken breast tissues for two sets of data (at 125 mV with an exposure time of 0.8 and 1.2 s) as shown in Fig. 4(c1–1). The fitting performance of single-phase exponential functions with the recovery segments in chicken breast does not exhibit the same level of accuracy as observed in porcine tissues. This disparity in fitting quality can be attributed to the extended fitting time interval used for the chicken model (4 s) in comparison with that of porcine tissues (0.8 s), potentially introducing additional noisy data points from the plateau level. An extended temporal interval of 6 s in Figs. 4(c2–1), 4(c2–2), and 5(c2) was chosen to accommodate a two-phase exponential model. Therefore, two time constants that can be fitted for each recovery period are denoted as *τ*_fast_ (i.e., the smaller time constant) and *τ*_slow_ (i.e., the larger time constant), respectively. The results are listed in Table I. This selection aimed to offer a more comprehensive insight into the slower exponential elements and to prevent data loss. It is worth noting that this approach, while potentially introducing fitting complexity, resulted in a marginal improvement in R² when compared to the one-phase exponential model, even during prolonged fitting durations, for both tissue samples. When the applied voltage is relatively low or the ultrasonic exposure is short (e.g., 125 mV or 800 ms), the calculated fluorescence vs time profile is also nosier. This may explain why smaller R^2^ values were noticed in 125 mV, 800 ms; 125 mV, 1200 ms; and 145 mV, 800 ms groups, as shown in Fig. 5(c1–2).

**TABLE I.** The summary of best-fitting results in porcine and chicken tissues.

Tissue	FU Parameters		Reduction		Recovery							
			Linear		Single-phase		Two-phase				*M*-model	
	Voltage (mV)	Time (ms)	Slope	R^2^	*τ*_r_	R^2^	*τ*_slow_	*τ*_fast_	Fast%	R^2^	*M*	R^2^
Chicken	125	800	−0.49	0.97	1.53	0.88	17.89	0.54	21.11	0.91	0.0054	0.89
	125	1200	−0.41	0.96	1.42	0.85	18.28	0.57		0.87	0.0051	0.87
	125	1600	−0.38	0.97	2.41	0.93	5.61	0.41		0.95	0.0058	0.97
	145	800	−0.69	0.95	0.97	0.91	1.34	0.09		0.9	0.0079	0.83
	145	1200	−0.68	0.97	1.17	0.97	1.77	0.29		0.97	0.0078	0.92
	145	1600	−0.61	0.98	1.36	0.97	2.77	0.25		0.98	0.0063	0.92
Porcine	125	800	−0.98	0.91	0.12	0.95	0.71	0.08	63.26	0.95	0.069	0.90
	125	1200	−0.75	0.91	0.12	0.96	0.98	0.08		0.98	0.059	0.92
	125	1600	−0.50	0.93	0.16	0.96	1.47	0.13		0.98	0.050	0.96
	145	800	−1.13	0.92	0.12	0.95	0.70	0.08		0.96	0.041	0.88
	145	1200	−0.77	0.94	0.17	0.95	1.23	0.13		0.97	0.049	0.89
	145	1600	−0.53	0.96	0.15	0.95	2.05	0.15		0.96	0.069	0.90

Comparing the exponential fitting models, the fitting to the analytical solution for extracting the parameter M shows relatively lower R² (several groups provide R² between 0.83 and 0.9) as shown in Figs. 4(c3) and 5(c3), despite its inherent simplicity with only one parameter. This is understandable because the solution is an approximation (either a more accurate analytical solution or adopting a numerical solution with known other parameters should be developed in the future). While keeping this disadvantage in mind, *M* is 0.056 ± 0.011 (mm^2^/s) in porcine tissues, significantly higher than *M *= 0.0064 ± 0.0012 (mm^2^/s) obtained with chicken breast. The fitted *M* shows not only a relatively stable value for different experimental conditions (∼19%–20% variations, i.e., = 0.011/0.056 and 0.0012/0.0064) but also shows significantly different values for the two types of tissues (∼8.75 times, i.e., = 0.056/0.0064). These results indicate that the nanoparticle has higher transportability in the porcine tissue sample than in the chicken breast tissue samples adopted in this study. Meanwhile, the effect of the experimental conditions on *M* is much less significant than the tissue types used in this study.

Table I summarizes the fitting parameters obtained with different equations. The 125 mV group has a lower absolute slope compared to the result for 145 mV group (−0.43 ± 0.06 vs −0.74 ± 0.24). During the ultrasound exposure, the displacements of nanoparticles were mainly dependent on the ultrasound strength. Therefore, a higher applied ultrasonic voltage means a more significant streaming effect in the tissues, leading to a faster fluorescence reduction rate. Fitting recovery data to a single exponential equation yields an average exponential time constant *τ*_r_ = 0.14 ± 0.02 in porcine tissues and *τ*_r_ = 1.48 ± 0.50 in chicken tissues. The bi-exponential regression models give the fast (*τ*_fast_) and slow (*τ*_slow_) time constants and the relevant percent of the fast decay component (Fast%). It is notable that these time constants in the bi-phase exponential fitting exhibit distinct values in both pork and chicken tissues (*τ*_slow _= 1.19 ± 0.52 vs 7.94 ± 8.00; *τ*_fast _= 0.11 ± 0.03 vs 0.36 ± 0.18). In summary, the three time constants (*τ*_r_, *τ*_slow_, and *τ*_fast_) are tissue-dependent. The porcine muscle tissues showed smaller values than the chicken breast tissues, which indicates that the nanoagents (SR101-PLGA) in porcine tissues sample should be transported relatively easier and quicker. The shared percentage of fast exponential component (Fast%) in porcine is higher than that in chicken (63.26 vs 21.11). Therefore, the porcine tissues exhibit notably shorter relaxation times, accompanied by a higher proportion of rapid change, which can be attributed to the faster fluid flow through larger pores interspersed among pork muscle fibers (see Fig. 6).

In both tissue samples, the value of *τ*_r_ seems to be not correlated with the exposure time. *τ*_slow_ in porcine tissues increases with the exposure time, but it is not in chicken breast tissues (especially when the voltage is 125 mV). Additionally, there is not an obvious relationship between *τ*_slow_ and the voltage. No obvious relationship can be found between *τ*_fast_ and the two experimental parameters (i.e., voltage and exposure time). This is also true for the relationship between the parameter *M* and the two experimental parameters, which may be an indication that the transportability is mainly dependent on particles and tissues, not experimental parameters. We defined *M* with tissue permeability *k*, tissue fluid viscosity μ, the retardation factor $R_{f}$ between interstitial fluid and the agent, and tissue apparent modulus *H*. As a result, we do not consider that there should be any correlation between *M* value and acoustic amplitude or exposure time if there is no significant heat-induced change to tissue porosity, elasticity, and tissue fluid viscosity during ultrasound irradiation. On the other hand, increasing ultrasound amplitude and exposure time will increase the flow velocity and nanoagent concentration change, but this does not affect the transportability *M*. Currently, it is unclear how to understand the relationships between the time constants and the experimental parameters because the physical meanings of these time constants (*τ*_r_, *τ*_slow_, and *τ*_fast_) are not as clear as the parameter *M*, so further studies should be conducted in future.

### C. Limitations and future work

This study conducted a detailed experimental investigation into the interactions between FU and nanoparticles, elucidating the experimental findings through SIF-TUM. However, the tissue medium was simplistically characterized as a two-phase mixture comprising solid and liquid matrices. In reality, biological tissues possess a far more complex structure, with the presence of a vascular network, osmotic pressures, and various tissue heterogeneities, leading to the non-uniform distribution of nanoparticles.^26,27^ In this analysis, only dominant bioeffects and tissue properties were considered to maintain model simplicity and facilitate comprehension. Nevertheless, it is important to notice that future research endeavors may need to incorporate additional factors to explain the experimental results in a detailed way. Future *in vivo* animal models can also be considered for assessing the ultrasound-mediated transport of nanoparticles in the presence of blood clearance, vascular permeability, and lymphatic drainage.^28,29^ Furthermore, the fitting process was restricted to only one or two parameters to facilitate the analysis and visualization of data trends. Extending the current fitting algorithm with multiple parameters may improve the accuracy.

Another future work could be exploring the effects of the size of nanoparticles. In our simulation model,^14^ nanoagents with an average size smaller than that of tissue pore size should be able to pass through the pores without significant resistance. Agents with a diameter between 10 and 40 nm achieve the optimized concentration reduction when the average diameter of the tissue pores is 80 nm. This means that within this diameter range, the agents can freely pass through the tissue pores and avoid significant natural diffusion due to their medium size. When the diameter is larger than 40 nm, the resistance of tissue to the agent transport becomes significant, and the concentration change becomes difficult. On the other hand, when the diameter is smaller than 10 nm, although these agents can easily pass through the pores, they can also diffuse back into the focal volume more efficiently than the larger ones, which reduces the efficiency of concentration change.

## IV. CONCLUSION

In conclusion, this study experimentally demonstrated the feasibility of using ultrasound to induce nanoparticle motions via interstitial fluid streaming and tissue recovery within biological tissues. The dynamics of the fluorescence reduction and recovery induced by the SIF-TUM effect during and after ultrasound exposure were monitored and analyzed via three models: single-phase exponential regression, two-phase exponential regression, and an approximated analytical solution of the SIF-TUM model via the parameter of *M*. To avoid the thermal effect, a temperature-insensitive nanoparticle, SR101-PLGA, was synthesized and adopted in this study. In both chicken breast and porcine muscle tissues, we have successfully observed the reduction and recovery of the fluorescence induced by ultrasound, which agrees with the prediction of the SIF-TUM models. The fluorescence reduction period and extent were significantly influenced by the ultrasound pressure and exposure time in chicken breast tissues, which agree with the results from the SIF-TUM models. In addition, all the time constants (*τ*_r_, *τ*_slow_, and *τ*_fast_) depend on tissue type. Compared with chicken breast tissues, porcine muscle tissues seem more favorable for the nanoagents (SR101-PLGA) to transport. The parameter of *M*, which is defined as the transportability of the nanoagent in tissues via the SIF-TUM models, was found independent of ultrasound experimental parameters but was highly related to tissue types. Porcine muscle tissues adopted in this study showed much higher (∼8.75 times) *M* values than those in chicken breast tissues. Therefore, it indicates that the transportability of the adopted nanoagent (SR101-PLGA) is much higher in porcine muscle tissues than in chicken breast tissues. The study underscores the potential significance of this research in advancing our understanding of ultrasound-involved nanoparticle delivery, which may enhance drug delivery efficiency for both therapeutic and diagnostic applications.

## Data Availability

The data that support the findings of this study are available from the authors upon reasonable request.
